# Phenotypic Plasticity Beyond Domestication: Trait- and Accession-Dependent Responses to Light in *Capsicum annuum*

**DOI:** 10.3390/plants15182825

**Published:** 2026-09-15

**Authors:** Virginia Solís-Montero, Emil O. Téllez-Villagómez, Miguel A. Munguía-Rosas, Rafael Bello-Bedoy

**Affiliations:** 1 Facultad de Ciencias, Universidad Autónoma de Baja California, Carretera Transpeninsular Ensenada-Tijuana No. 3917, Col. Playitas, Ensenada C.P. 22860, Mexico; virginiasols082@gmail.com (V.S.-M.); emil.tellez@uabc.edu.mx (E.O.T.-V.); 2 Laboratorio de Ecología Terrestre, Centro de Investigación y de Estudios Avanzados del Instituto Politécnico Nacional (Cinvestav), Km 6 Antigua Carretera a Progreso, Mérida C.P. 97203, Mexico; munguiarma@cinvestav.mx

**Keywords:** artificial selection, *Capsicum annuum* var. *glabriusculum*, crop–wild system, domestication, light gradient, reaction norms, shade response, vegetative traits

## Abstract

Domestication may modify phenotypic plasticity by shifting plants from heterogeneous natural habitats to more uniform agricultural environments, potentially reducing environmental responsiveness. However, whether domestication consistently alters plasticity across traits, populations, and varieties remains unresolved. Here, we evaluated phenotypic plasticity to light availability in a crop–wild system of *Capsicum annuum* by comparing three wild populations and three domesticated accessions grown under full, moderate, and low light conditions (~1000, 565, and 160 µmol m^−2^ s^−1^, respectively). We quantified vegetative, physiological, and reproductive traits and assessed plasticity using hierarchical models, reaction norm slopes, and multivariate ordination. Light availability strongly influenced plant phenotype, but the effects of domestication were weak and not uniform across varieties and traits. Vegetative traits including leaf area, specific leaf area, leaf number, and leaf mass showed pronounced responses to light availability, whereas flowering-related traits were less consistently responsive. Low light induced the clearest multivariate phenotypic shift, characterized by larger and thinner leaves. However, these vegetative adjustments did not maintain reproductive performance. Fruit production remained relatively stable under medium light but declined sharply under severe shade. Together, our results show that domestication does not lead to a uniform reduction in phenotypic plasticity. Instead, plasticity in chili depends on the trait and on the population or variety evaluated and is expressed more strongly in vegetative than in reproductive components. These findings indicate that the biological consequences of plastic responses should be evaluated in relation to reproductive performance, not only vegetative adjustment.

## 1. Introduction

Domestication has produced pronounced genomic and phenotypic divergence between cultivated plants and their wild relatives [1,2,3], reflecting the cumulative effects of sustained artificial and unconscious human selection on traits such as plant growth and architecture, fruit size, and metabolic profiles associated with taste and use [4,5,6,7,8]. However, domestication also involves a transition from heterogeneous natural habitats to more uniform managed environments. These conditions represent a distinct ecological context in which water, nutrients, light, competition, and biotic interactions are often modified by human management [5,9]. Thus, domestication may influence not only the visible traits selected by humans, but also integrated functional responses related to growth, reproduction, and environmental responsiveness.

Phenotypic plasticity is one functional response that may be altered by this ecological transition. Plasticity is defined as the capacity of a genotype to produce different phenotypes across environmental conditions, and it is commonly described through reaction norms across environmental gradients [10,11,12]. Environmental heterogeneity may maintain plastic responses that allow plants to adjust growth, development, and reproduction under variable conditions, whereas more homogeneous agricultural environments may favor predictable performance and reduced responsiveness to environmental cues [9,13,14]. However, plasticity is not expected to be expressed uniformly across the whole plant because traits differ in their developmental regulation, timing, and sensitivity to environmental signals [14,15,16]. In addition, selection during domestication has strongly focused on productivity, particularly fruit production, which may also affect other plant functions. Thus, domestication may generate trait-specific patterns of plasticity, with some traits showing altered or reduced responses and others retaining plasticity because of their role in survival, growth, and reproduction.

Among abiotic factors, light availability often differs substantially between natural and agricultural systems and strongly influences plant traits associated with each environment [17,18]. In natural habitats, plants typically experience highly variable light conditions, ranging from full sun exposure to deep shade under the canopy [19]. In contrast, agricultural systems are often managed to reduce shading among individuals in order to maximize light interception and photosynthetic performance [20]. This shift in light environment represents one ecological component of domestication and may have contributed to morphological, physiological, and biochemical differences between wild and cultivated plants [5]. Thus, changes in light availability associated with domestication may have important evolutionary consequences because selection under heterogeneous natural habitats may maintain plastic responses to shade, whereas more uniform agricultural environments may reduce the advantage of responding plastically to variation in light.

Despite the relevance of phenotypic plasticity for understanding the evolutionary consequences of domestication, relatively few experimental studies have directly compared plastic responses between crops and their wild progenitors under controlled contrasting environments [14,15,16]. Matesanz and Milla [14] evaluated responses of seven cultivars to variation in water and nutrient availability and reported substantial variation in plastic responses among environments and morpho-functional traits, indicating that domestication does not necessarily lead to a general reduction in plasticity. Similarly, Munguía-Rosas [16] compared wild and domesticated forms of *Cnidoscolus* under contrasting light environments and found greater plasticity in stem and leaf traits in wild plants than in domesticated plants. In both studies, reaction norms varied among traits and plant groups, suggesting that domestication does not affect plasticity uniformly across the plant phenotype. Although these studies provided relevant information to begin understanding plastic responses in domesticated plants, direct comparisons of plasticity between wild progenitors and domesticated forms within the same lineage remain limited. Such comparisons can reveal whether differences in plasticity vary among traits and whether plastic responses influence plant fitness, reproduction, and yield.

*Capsicum annuum* var. *glabriusculum*, the wild progenitor of most Mexican cultivated chili varieties, is a long-lived perennial shrub that reaches 2 m in height and produces several hermaphroditic flowers and fruits from June to October [21,22,23,24]. Domestication began in northern and central Mexico approximately 6400 years BP [25], producing numerous cultivated forms with contrasting phenotypes. Wild populations occur mainly in tropical dry forests [26], where plants experience heterogeneous light environments ranging from shaded understory to full sun exposure [27,28]. In these environments, light availability strongly influences plastic responses in growth, physiological traits, and fruit production [29], although the magnitude of these responses varies among traits and populations. Light limitation commonly reduces plant growth and fruit production, indicating that light availability affects both vegetative performance and reproductive output [22,30]. Cultivated varieties, in contrast, have evolved under agricultural environments commonly characterized by high light availability, which may relax selective pressures for maintaining plasticity. Nevertheless, cultivated *Capsicum* varieties retain substantial phenotypic responses to environmental variation, including shading and water limitation [31,32,33]. Although previous studies have examined plastic responses to light variation within and among populations of *C. annuum* [29,31], direct comparisons of phenotypic plasticity between wild and domesticated forms remain limited. In addition, a previous study found that domesticated plants exhibited reduced resistance to herbivores [34], raising the possibility that domestication may also alter other functional responses, including phenotypic plasticity. Comparisons within this crop–wild system can therefore reveal whether domestication has modified plastic responses to light and whether these responses differ among functional traits.

Here, we examined whether domestication has altered phenotypic plasticity to light availability in a crop-wild system of Mexican chilies *Capsicum annuum*. We grew plants from three wild populations and three domesticated varieties under three contrasting light environments (full light, moderate shade, and severe shade) and measured vegetative (stem height, stem diameter, and leaf traits), physiological (chlorophyll content), and reproductive traits (height to first flower, days to flowering, and fruit number). Specifically, we asked whether wild populations and domesticated varieties differ in their plastic responses to light and whether these responses vary among functional traits. Because wild chilies typically occur in a heterogeneous matrix of open and partially shaded habitats, whereas domesticated varieties have been improved under open-field conditions with high light availability, we expected domestication to influence plastic responses to light, but that the magnitude and direction of these effects would differ among traits and among populations and varieties. We expected particularly marked responses in vegetative traits because they are closely related to light capture, growth, and plant performance under shade. These expectations are consistent with hypotheses in the domestication literature suggesting that selection during domestication may influence environmental responsiveness while generating trait-specific patterns of plasticity depending on the function and selective history of each trait [9,14,35].

## 2. Results

### 2.1. General Patterns

Light availability affected multiple phenotypic traits, whereas the effects of domestication status were not uniform across the phenotypes (Table 1). Nested ANOVA analyses showed significant differences among the evaluated wild populations and domesticated accessions for several traits. Accession-specific responses to light were also detected for leaf area, fresh leaf mass, dry leaf mass, and chlorophyll content. The count models further indicated heterogeneous responses in leaf number and fruit production. Overall, phenotypic plasticity depended on the trait evaluated and on the identity of the accession rather than reflecting a uniform domestication effect.

### 2.2. Vegetative Traits

**Figure 1:**
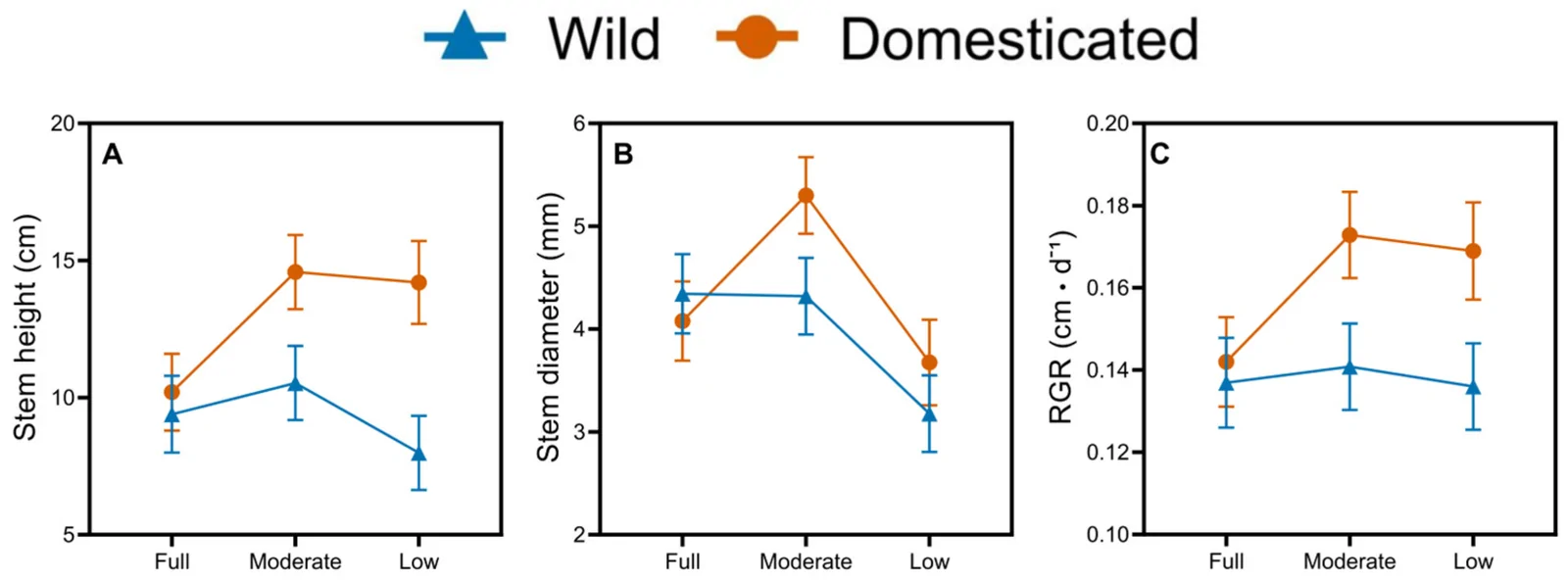
Reaction norms of stem-related traits in wild populations and domesticated varieties of *Capsicum annuum* grown under three light environments. Panels show (**A**) stem height, (**B**) stem diameter, and (**C**) relative growth rate (RGR). Stem height and diameter correspond to measurements taken at 57 days after germination. Blue triangles represent wild populations and orange circles represent domesticated varieties. Points indicate means and error bars represent ± SE. Full, medium, and low light correspond to approximately 1000, 565, and 160 µmol m^−2^ s^−1^, respectively. The values shown within each panel correspond to the *p* value of the domestication status × light interaction.

**Figure 2:**
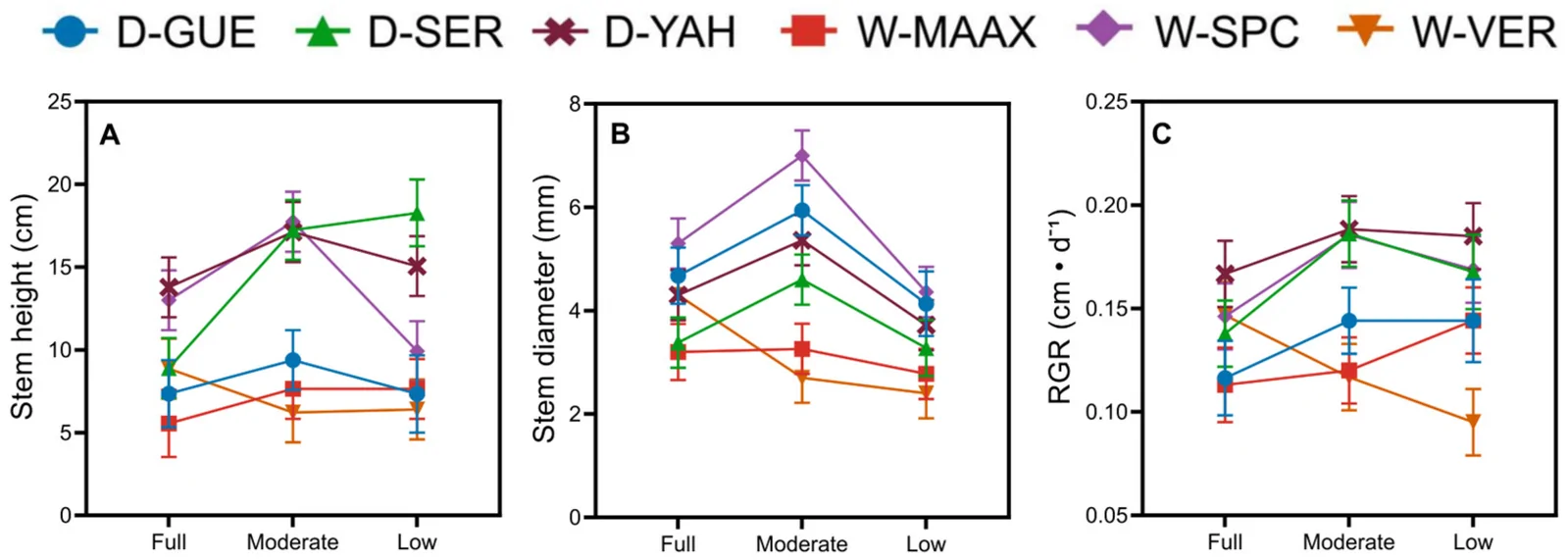
Reaction norms of stem-related traits among wild populations and domesticated varieties of *Capsicum annuum* grown under three light environments. Panels show (**A**) stem height, (**B**) stem diameter, and (**C**) relative growth rate (RGR). Stem height and diameter correspond to measurements taken at 57 days after germination. Each line represents one population or variety. D-GUE, D-SER, and D-YAH correspond to the domesticated varieties Güero, Serrano, and Yahualica, respectively. W-MAAX, W-SPC, and W-VER correspond to the wild populations MAAX, SPC, and VER, respectively. Points indicate means and error bars represent ± SE. Full, medium, and low light correspond to approximately 1000, 565, and 160 µmol m^−2^ s^−1^, respectively.

Light intensity significantly affected stem diameter, leaf area, and specific leaf area (SLA) (Table 1). Stem diameter was highest under medium light and lowest under low light, increasing by 14.3% from full to moderate light and then decreasing by 29.3% from moderate to low light. Stem height and stem diameter also differed significantly among accessions nested within domestication status. In contrast, stem height and relative growth rate (RGR) did not show significant overall responses to light in the hierarchical models (Figure 1 and Figure 2). RGR showed no significant effects of accession nested within domestication status or light × accession nested within domestication status.

**Figure 3:**
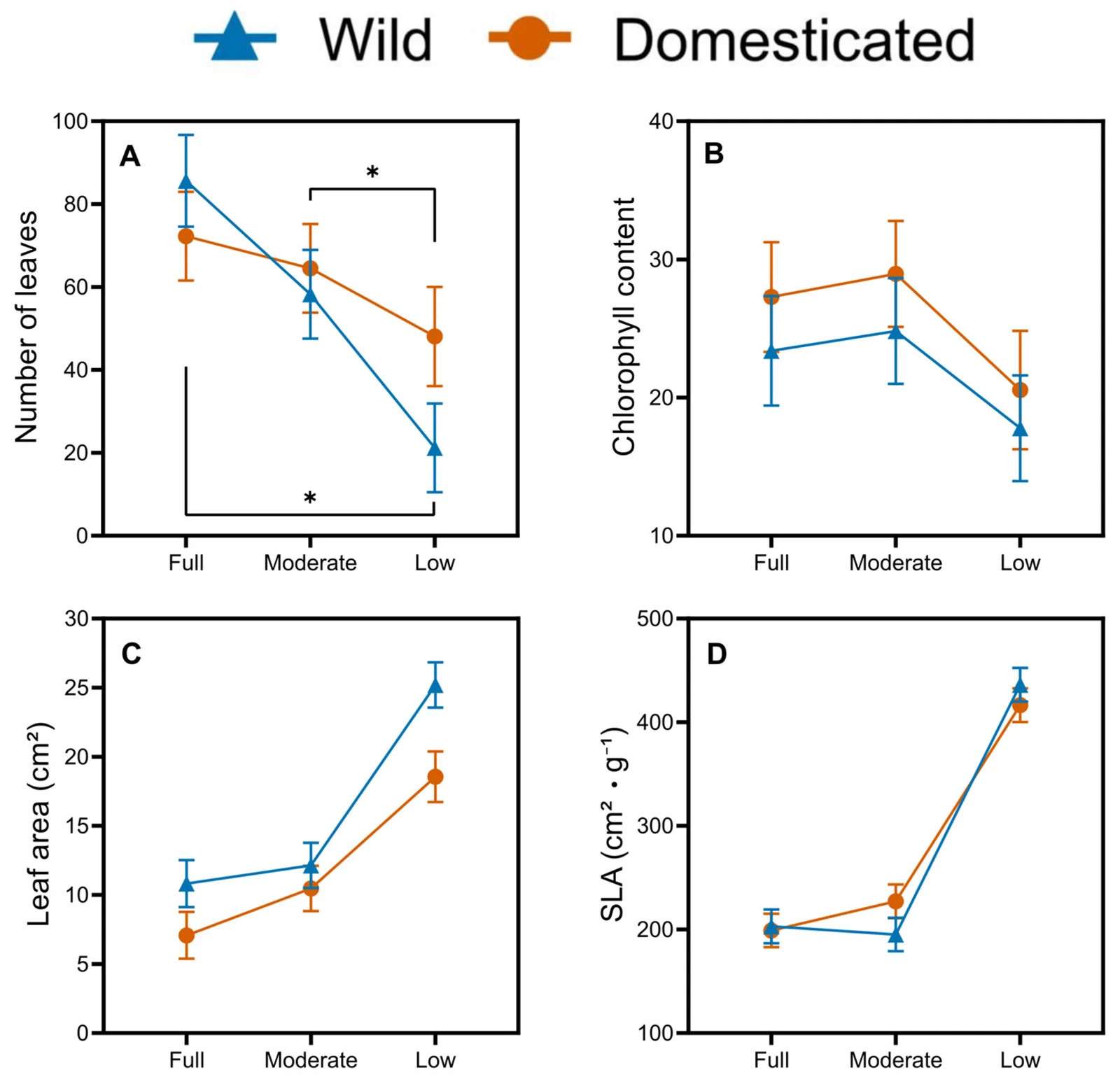
Reaction norms of leaf-related traits in wild and domesticated accessions of *Capsicum annuum* grown under three light environments. Panels show (**A**) number of leaves, (**B**) chlorophyll content, (**C**) leaf area, and (**D**) specific leaf area (SLA). Blue triangles represent wild populations and orange circles represent domesticated varieties. Points indicate means and error bars represent ± SE. Full, medium, and low light correspond to approximately 1000, 565, and 160 µmol m^−2^ s^−1^, respectively. Connecting brackets and * indicate significant pairwise differences between light treatments based on Tukey’s HSD test (*p* < 0.05).

**Figure 4:**
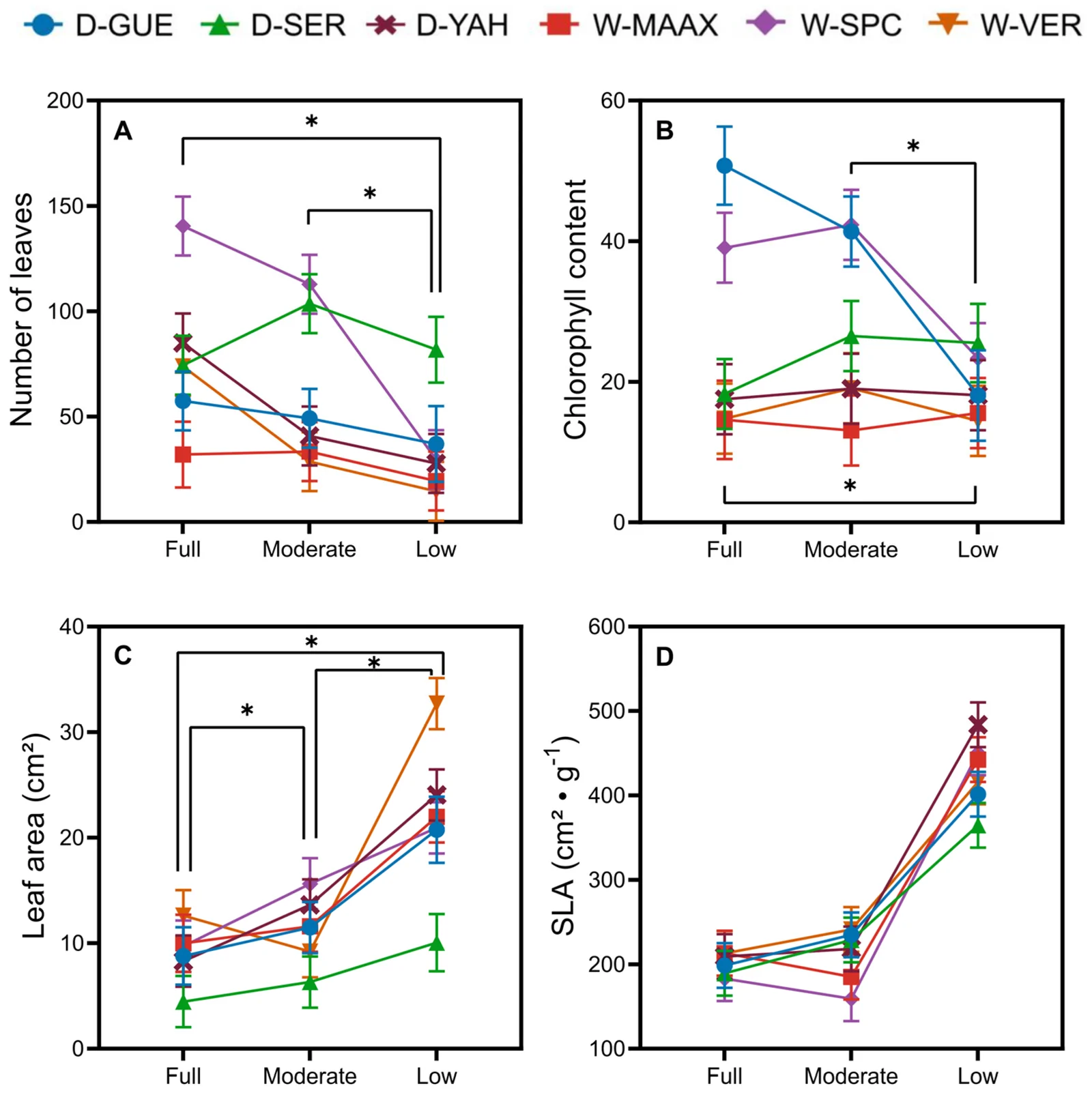
Reaction norms of leaf-related traits among wild and domesticated accessions of *Capsicum annuum* grown under three light environments. Panels show (**A**) number of leaves, (**B**) chlorophyll content, (**C**) leaf area, and (**D**) specific leaf area (SLA). Leaf counting and measurements were taken 97 days after germination at the end of the experiment. Each line represents one population or variety. D-GUE, D-SER, and D-YAH correspond to the domesticated varieties Güero, Serrano, and Yahualica, respectively. W-MAAX, W-SPC, and W-VER correspond to the wild populations MAAX, SPC, and VER, respectively. Points indicate means and error bars represent ± SE. Full, medium, and low light correspond to approximately 1000, 565, and 160 µmol m^−2^ s^−1^, respectively. Connecting brackets and * indicate significant pairwise differences between light treatments based on Tukey’s HSD test (*p* < 0.05).

Mean leaf area increased from 8.95 cm^2^ under full light to 22.24 cm^2^ under low light, representing a 148.5% increase, whereas SLA increased from 201.01 to 426.36 cm^2^ g^−1^, representing a 112.1% increase, indicating the production of larger and thinner leaves under severe shade. For leaf area, this response differed among accessions, as indicated by the significant light × accession nested within domestication status interaction. Leaf number declined from an average of 78.72 leaves under full light to 33.15 leaves under low light, representing a 57.9% increase. Leaf number was significantly affected by light treatment, domestication status × light. In addition, accession nested within domestication status, and light × accession nested within domestication status were significant (Table 1; Figure 3 and Figure 4). Chlorophyll content also varied significantly among accessions, and its response to light differed significantly among accessions, as indicated by the interaction light × accession nested within domestication status (Figure 3 and Figure 4). Together, these results indicate that vegetative responses to light were trait-specific and heterogeneous among the populations and varieties evaluated.

### 2.3. Reproductive Traits

**Figure 5:**
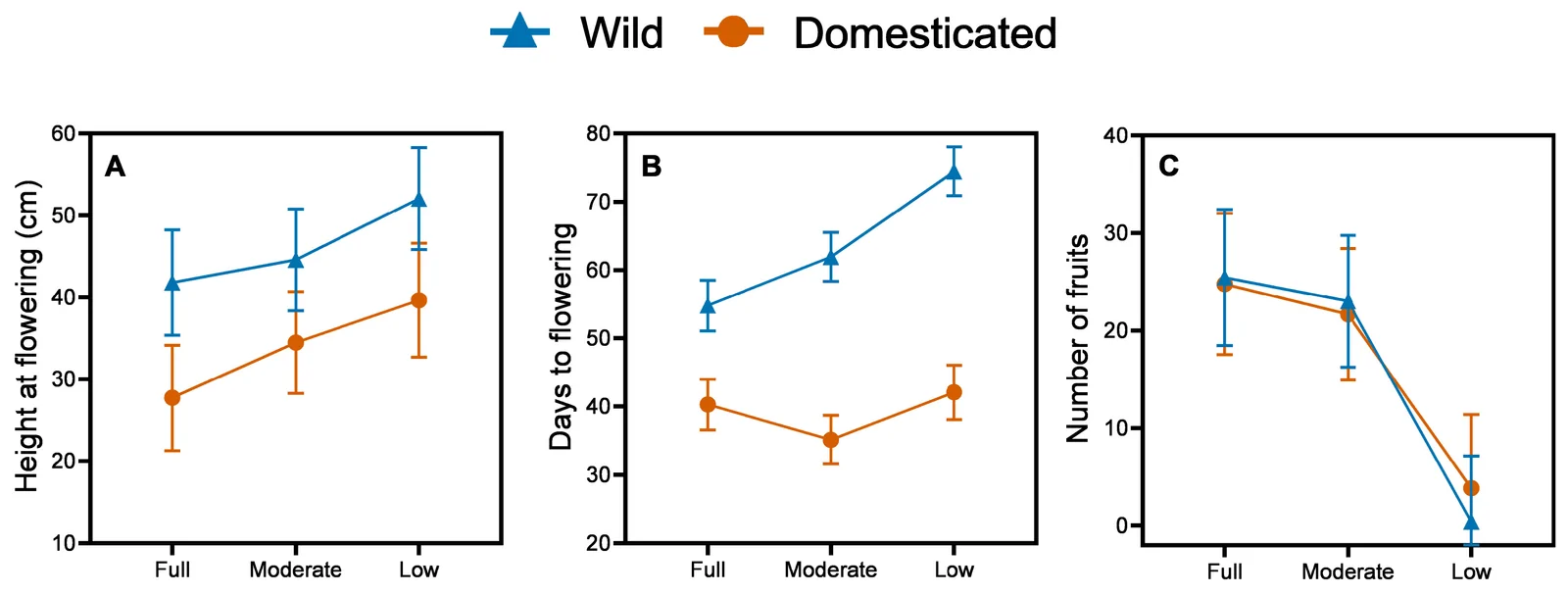
Reaction norms of reproductive traits in wild and domesticated accessions of *Capsicum annuum* grown under three light environments. Panels show (**A**) height to first flower, (**B**) days to flowering, and (**C**) number of fruits per plant. Blue triangles represent wild populations and orange circles represent domesticated varieties. Points indicate means and error bars represent ± SE. Full, medium, and low light correspond to approximately 1000, 565, and 160 µmol m^−2^ s^−1^, respectively. The values shown within each panel correspond to the *p* value of the domestication status × light interaction.

**Figure 6:**
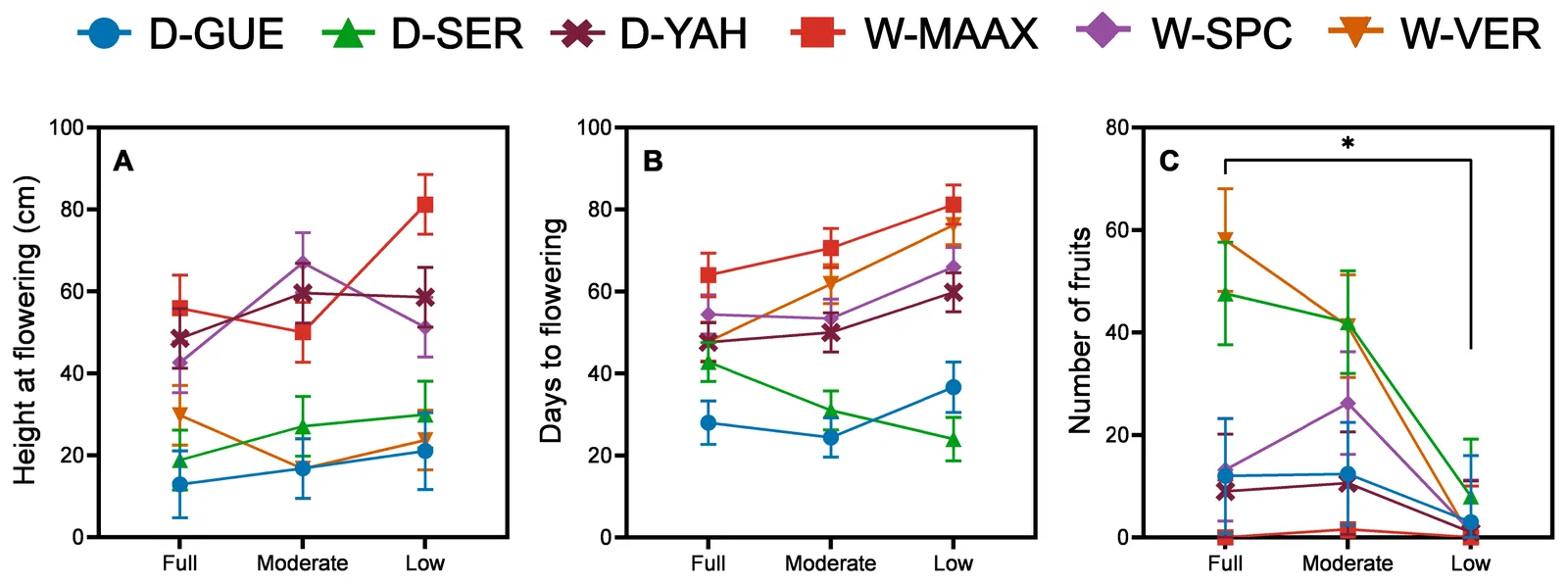
Reaction norms of reproductive traits among wild and domesticated accessions of *Capsicum annuum* grown under three light environments. Panels show (**A**) height to first flower, (**B**) days to flowering, and (**C**) number of fruits per plant. Each line represents one population or variety. D-GUE, D-SER, and D-YAH correspond to the domesticated varieties Güero, Serrano, and Yahualica, respectively. W-MAAX, W-SPC, and W-VER correspond to the wild populations MAAX, SPC, and VER, respectively. Points indicate means and error bars represent ± SE. Full, medium, and low light correspond to approximately 1000, 565, and 160 µmol m^−2^ s^−1^, respectively. Connecting brackets and * indicate significant pairwise differences between light treatments based on Tukey’s HSD test (*p* < 0.05).

Height to first flower and days to flowering differed among wild and domesticated accessions, but neither trait showed a significant overall response to light in the nested ANOVA analyses (Table 1). Fruit number was strongly affected by light availability. Observed mean fruit production remained relatively stable between full and medium light (25.11 and 22.33 fruits per plant, respectively) but declined to 1.93 fruits per plant under low light, representing a reduction of approximately 92% relative to full light. The analyses detected significant effects of domestication status and light treatment, whereas the domestication status × light interaction was not significant (Table 2). In contrast, the population or variety × light interaction was significant, indicating that the magnitude of the reproductive response differed among the six groups evaluated (Figure 5 and Figure 6).

### 2.4. Multivariate Analysis (Procrustes ANOVA and Phenotype Trajectory Analysis [PTA])

**Figure 7:**
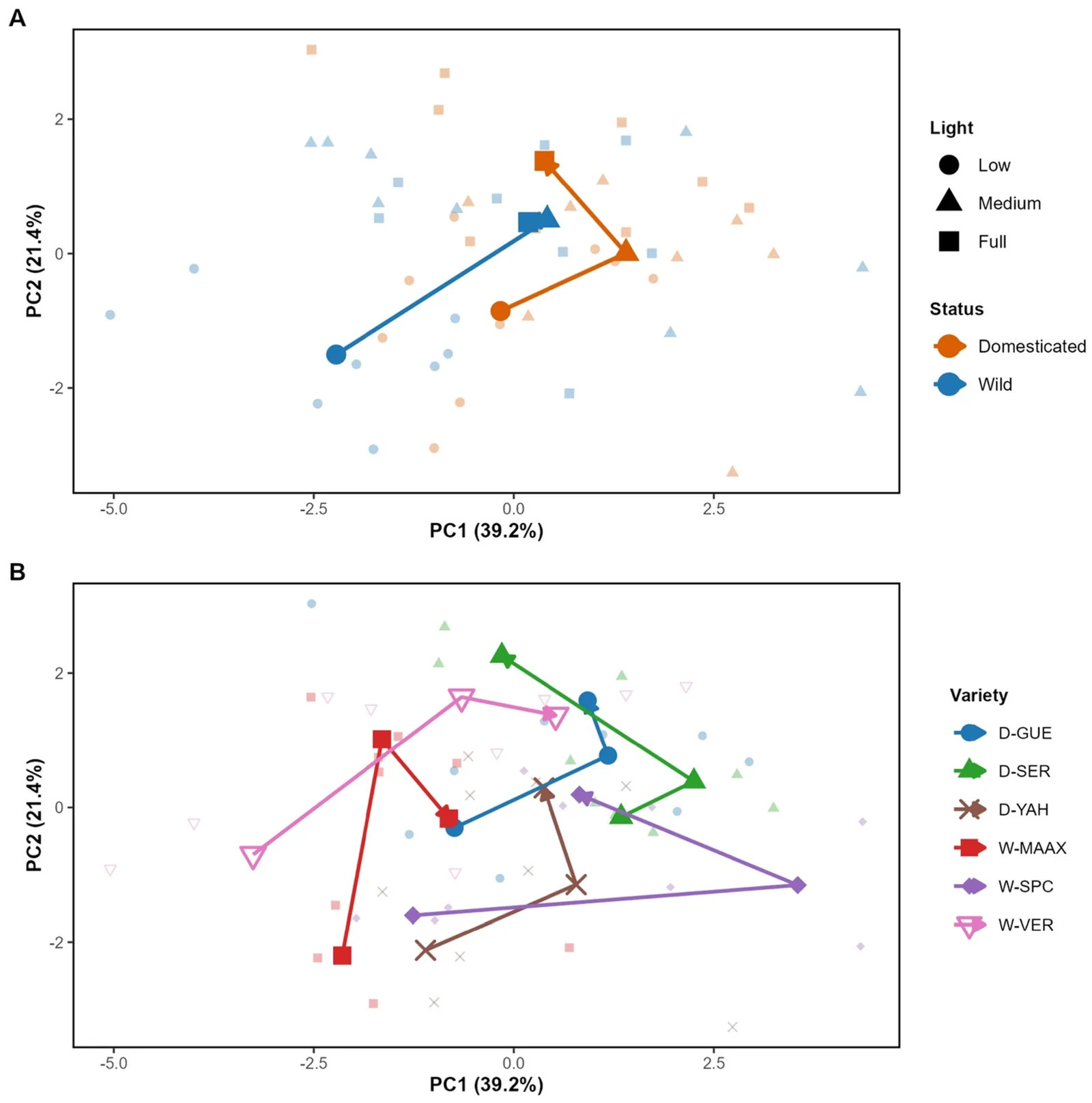
Multivariate phenotypic trajectories of wild and domesticated Capsicum annuum across three light environments. (**A**) Trajectories according to domestication status. Orange symbols and lines represent domesticated varieties, whereas blue symbols and lines represent wild populations. Symbol shape indicates light environment: circles = low light, triangles = medium light, and squares = full light. (**B**) Trajectories for the six individual accessions, each represented by a unique color and symbol combination (D-GUE, D-SER, D-YAH, W-MAAX, W-SPC, and W-VER). In both panels, large symbols connected by lines represent multivariate group centroids across light environments, whereas semi-transparent background symbols represent individual plants. Percentages on the axes indicate the proportion of total variation explained by PC1 and PC2.

Procrustes ANOVA revealed significant main effects on multivariate morphospace position for both domestication status (*F* _1,48_ = 5.03, *p* < 0.01) and light treatment (*F* _2,48_ = 6.77, *p* < 0.01). However, the interaction term was not significant (*F* _2,48_ = 1.09, *p* = 0.36). Consistently, PTA showed no significant differences between domesticated and wild trajectories in total magnitude of plasticity (d = 0.13, Z = −1.33, *p* = 0.89), vector orientation/direction (angle = 0.59, Z = 0.45, *p* = 0.32), or trajectory shape (d = 0.39, Z = 1.57, *p* = 0.06) (Figure 7A). Procrustes ANOVA for plant varieties indicated significant main effects for variety (*F* _5,36_ = 6.85, *p* < 0.01) and light treatment (*F* _2,36_ = 10.90, *p* < 0.01), as well as a significant variety × light interaction (*F* _10,36_ = 1.87, *p* < 0.01). Pairwise PTA further identified specific geometric divergences among variety trajectories across the light environments. Specifically, regarding plasticity magnitude, SPC exhibited greater overall plasticity path length compared to GUE (d = 4.38, Z = 2.26, *p* < 0.01) and SER (d = 3.48, Z = 1.86, *p* = 0.03), while trajectory angles diverged significantly between SER and SPC (angle = 1.94, Z = 2.18, *p* = 0.02) and between SER and VER (angle = 1.54, Z = 1.76, *p* = 0.04) and trajectory shape differed markedly between SER and SPC (Z = 2.65, *p* < 0.01), MAAX and SER (Z = 2.10, *p* = 0.01), and SPC and VER (Z = 1.80, *p* = 0.04) (Figure 7B).

## 3. Discussion

Phenotypic plasticity is widely recognized as an important component of the evolutionary process because it allows organisms to adjust trait expression across environments and respond optimally under heterogeneous conditions [10,36]. In domesticated plants, understanding how artificial selection affects phenotypic plasticity is especially relevant because artificial selection can reduce genetic variation [37] and often favors uniformity and predictable performance under managed conditions, leading to the expectation that domestication may reduce phenotypic plasticity [9]. Our results, however, do not support a uniform reduction of plasticity across the study system. Instead, responses varied among traits and, even more strongly, among populations and varieties, indicating that (wild) population- and (domesticated) variety-specific histories were important components of the observed pattern. Both the univariate analyses and the multivariate PCA showed that the clearest phenotypic displacement occurred under low light, whereas plants grown under full and medium light were less different. Importantly, the vegetative response to low light was not accompanied by maintenance of fruit production, indicating that these responses were insufficient to preserve reproductive performance under strong shade [38]. Taken together, these results suggest that the effects of artificial selection during domestication on phenotypic plasticity depend on the function and selective history of individual traits.

Because relatively few studies have explicitly evaluated phenotypic plasticity within and across crop–wild systems, it remains unclear whether domestication consistently reduces environmental responsiveness across traits, populations, and varieties, as previously proposed [9,16]. In our study, plasticity did not decline uniformly with domestication. Domestication status × light interactions were generally weak or absent, indicating that wild populations and domesticated varieties did not differ consistently in their responses to the light gradient. In contrast, population or variety × light interactions were stronger for several traits, particularly vegetative traits, indicating that plastic responses depended more on the identity of the population or variety than on domestication status alone. The multivariate trajectory analysis reinforced this pattern, showing that wild and domesticated plants did not differ in the magnitude, direction, or shape of their phenotypic trajectories across light environments, whereas significant differences were detected among individual varieties (Figure 7). These results indicate that multivariate plastic responses were structured more strongly by variety identity than by domestication status. This pattern is consistent with previous studies showing that domestication does not alter plasticity uniformly across crops and their wild relatives, and that responses can vary substantially among cultivars and traits [14,16]. Similar heterogeneity has also been reported in *Capsicum annuum* and other crop–wild systems [32]. Likewise, cultivated forms of *Cnidoscolus aconitifolius* subjected to artificial selection on leaf and defensive traits retain substantial variation in plastic responses [16,39]. Thus, rather than supporting a general reduction in plasticity through domestication, our results indicate that phenotypic plasticity in chili is strongly influenced by population- and variety-specific histories, potentially reflecting differences in conscious and unconscious selection, environmental exposure, and local adaptation.

Interestingly, phenotypic plasticity was not expressed uniformly across traits, supporting the idea that different functional modules may respond differently to environmental variation [40]. A recent meta-analysis similarly showed that plastic responses are often trait-specific rather than organism-wide [41]. In our study, the clearest responses to the light gradient were observed in vegetative traits associated with plant architecture, growth, and leaf traits, which are closely related to shade acclimation [17]. Leaf area, number of leaves, chlorophyll content, and specific leaf area responded more strongly to variation in light availability than flowering-related traits. Leaf area is itself an integrated trait whose final size can result from variation in cell number, cell expansion, or both [32], and the regulatory hormonal mechanisms implicated in leaf growth. This pattern suggests that leaf- and growth-related modules were more responsive to light limitation than reproductive traits. Because light availability directly affects light interception and carbon gain, the uneven distribution of plasticity among traits likely reflects differences in the biological responses of functional modules. This interpretation is consistent with previous work showing that plastic responses depend on the environmental factor involved [38,42], as plants, including crops, may respond differently to variation in light, water, or nutrients [14]. To our knowledge, studies of plasticity in crop–wild systems have rarely evaluated vegetative and reproductive traits simultaneously, limiting broader inference about how plasticity is distributed across functional categories. Our results therefore do not support the existence of a single, uniform plastic syndrome in chili plants. Instead, they indicate that plasticity is trait-dependent and is expressed more strongly in vegetative than in reproductive components, particularly under severe light limitation.

A major result of our study is that low light induced the most pronounced phenotypic shift. Regardless of domestication status, plants grown under low light were clearly different, whereas those grown under full and medium light overlapped broadly. In our experiment, medium and low light reduced incident radiation by approximately 43.5% and 84%, respectively, relative to full-light conditions, indicating that the strongest phenotypic displacement occurred only under severe light limitation. This distinction is important because the medium-light treatment likely resembles the light environment experienced by some wild and cultivated landraces of chili peppers grown in backyard conditions [22,28], whereas the low-light treatment represents a much stronger constraint, such as those commonly found in the understory [19]. Plants grown under low light had a higher specific leaf area and leaf area, consistent with a shade-acclimation phenotype [17,18]. However, this vegetative response was not accompanied by maintenance of fruit production. Thus, although low light promoted a plastic adjustment in leaf morphology and growth-related traits, these responses were insufficient to prevent a decline in reproductive output.

Indeed, reaction norm slopes showed that plastic responses were minimal between full and medium light but increased markedly between medium and low light (Table S1). The strongest plastic responses were observed in specific leaf area, leaf area, number of leaves, and fruit number, whereas several traits showed weak or near-zero responses under moderate light reduction. Because not all phenotypic plasticity increases plant performance [43], the biological consequences of these responses must be evaluated in relation to reproduction. Under moderate shade, chili plants maintained relatively stable reproductive output. In contrast, severe light limitation triggered pronounced vegetative adjustments, including the production of larger and thinner leaves, but also caused a marked decline in fruit production. At the physiological level, reduced irradiance can limit photosynthetic carbon fixation and thereby decrease the amount of assimilated carbon available for growth and reproductive sinks such as developing fruits. Conversely, maintaining carbon assimilation under moderate shade could help sustain reproductive output. The decline in reproductive output observed in this study coincided with delayed flowering under severe shade, particularly in wild populations, whereas domesticated varieties flowered early, showing a weaker flowering response. Reproductive responses to shade can differ among species. Although shade commonly accelerates flowering in shade-intolerant annual plants, simulated and natural shade delayed flowering in *Medicago sativa*, where extending the vegetative phase may allow plants to accumulate reserves under suboptimal light conditions [44]. In chili, however, delayed flowering under severe shade was associated with reduced fruit production, suggesting that prolonging the vegetative phase was insufficient to preserve reproductive performance. Similar patterns have been reported in other species, where shade responses can improve performance under some conditions but become insufficient or costly under stronger light limitation [45,46,47]. Thus, in our study, the stronger vegetative plasticity expressed under severe shade was insufficient to maintain reproductive performance.

Our study evaluated whether domestication altered phenotypic plasticity to light availability in wild and domesticated *Capsicum annuum*, and whether these responses were expressed consistently across vegetative and reproductive traits and among populations and varieties. This study contributes to the limited number of crops–wild comparisons that evaluate vegetative and reproductive responses simultaneously. By doing so, it provides a broader view of how plasticity is distributed across functional trait categories and shows that the effects of domestication are neither uniform nor easily generalized. Instead, our results highlight the importance of population- and variety-specific plasticity and indicate that the limits of plastic responses must be evaluated in relation to reproductive performance, not only vegetative adjustment. More broadly, the variation in plastic responses observed among populations and varieties may be relevant for both evolutionary research and crop improvement under increasingly variable environmental conditions [9,20]. Our findings also suggest a potential practical implication: chili plants maintained relatively stable fruit production under moderate shade, indicating that cultivation under partial shade may be feasible without a detectable reproductive cost. This possibility may be relevant for farmers interested in using moderate shade to buffer crops against high temperatures associated with climate change [48]. However, field experiments are needed to determine whether moderate shading improves crop performance under combined abiotic and biotic stresses.

## 4. Materials and Methods

### 4.1. Plant Material and Growth Conditions

All plants used in this study were grown from seeds maintained in the germplasm repository of the Laboratory of Ecological Genetics and Evolution at the Universidad Autónoma de Baja California, Ensenada, Mexico. The plant material included three wild-derived accessions and three domesticated accessions of *Capsicum annuum*. Here, we use “accession” to refer to each wild-derived population or domesticated variety maintained in the germplasm repository and evaluated in the experiment. The wild-derived populations corresponded to *C. annuum* var. *glabriusculum* and originated from geographically distinct regions of Mexico: Sonora (SON), Veracruz (VER), and Yucatán (YUC). The domesticated varieties corresponded to *C. annuum* var. *annuum*. Güero (GUE) and Serrano (SER) were originally obtained from Hortaflor^®^ (Rancho Los Molinos, Morelos, Mexico) commercial seed packets, whereas Yahualica (YAH) was originally provided by the Regional Center for Integrated Services for Protected Agriculture (CRESIAP; Jalisco, Mexico). These domesticated varieties were subsequently maintained and propagated under greenhouse conditions in the germplasm repository. Starting material consisted of seeds obtained from self-fertilized greenhouse-grown maternal plants representing each wild population or domesticated variety.

In May 2022, fifty seeds per wild and domesticated accession were soaked in distilled water for 72 h and subsequently treated with gibberellic acid (500 ppm) for 24 h to promote germination. Seeds were sown in 250 mL pots containing sterile Berger BM2 peat substrate (Berger, Saint-Modeste, QC, Canada) and maintained at 24–29 °C under controlled conditions. In June 2022, seedlings at the second true-leaf stage were transplanted into 2.5 L pots containing the same substrate. Roots and stems were buried up to the cotyledons to standardize early developmental conditions among individuals. After transplanting, plants were fertilized weekly with a 19–19–19 NPK solution (3 g L^−1^) and irrigated every other day to maintain substrate moisture and prevent water limitation.

### 4.2. Light Intensity Treatment

The experiment was conducted in a greenhouse under natural photoperiod conditions. Temperature was not experimentally controlled and varied with natural seasonal conditions, with mean temperatures ranging from 24 to 28 °C during the experimental period. Relative humidity was not controlled. Plants were exposed to three light environments established using shade cloth: full light (~1000 µmol m^−2^ s^−1^), medium light (~565 µmol m^−2^ s^−1^), and low light (~160 µmol m^−2^ s^−1^). Photosynthetic photon flux density was measured weekly at 12:00 h using an Apogee MQ-500 full-spectrum quantum meter (Apogee Instruments, Logan, UT, USA). The reported values correspond to the mean irradiance recorded for each treatment during the experiment. Full light represented conditions typical of open agricultural environments; the medium-light treatment represented an intermediate irradiance level resembling natural understory conditions, whereas the low-light treatment imposed severe light limitation [25,28]. Five biological replicates were assigned to each population or variety × light treatment combination (*n* = 90 plants). Plants were randomly assigned to treatments and periodically rotated within each light environment to reduce positional effects.

### 4.3. Trait Measurements

We measured vegetative, physiological, and reproductive traits to evaluate phenotypic responses to light availability. Stem height (cm) was measured from the base of the stem to the apical meristem using a flexible measuring tape. Stem diameter (mm) was measured at the base of each plant using a digital caliper. Relative growth rate (RGR, day^−1^) was calculated between weeks 4 and 6, corresponding to the onset of the shade treatments and a juvenile developmental stage before branching, using the standard logarithmic growth equation:

$$RGR=\frac{\left[ln(Hf)-ln(Hi)\right]}{\;(t2-t1)}$$

where *Hf* and *Hi* are final and initial stem height, respectively, and *t*2 − *t*1 is the time interval between measurements. Leaf number was recorded excluding senescent leaves.

Leaf area was quantified from ten fully expanded leaves per plant. Leaves were scanned, and individual leaf area was measured using WinFOLIA 16 image-analysis software (Regent Instruments Inc., Québec, QC, Canada). Fresh leaf mass, dry leaf mass, and specific leaf area (SLA) were quantified in a subsample of three plants per population or variety × light treatment combination (*n* = 54 plants). To determine fresh mass for this subsample, the same leaves used for leaf-area measurements were weighed immediately after collection to determine fresh mass using an MS204 precision balance (Mettler Toledo, Greifensee, Switzerland) to the nearest 0.0001 g. Leaves were subsequently dried at 65 °C for five days in a VWR drying oven and weighed again to determine dry mass. SLA (cm^2^ g^−1^) was calculated as leaf area divided by dry mass. Leaf mass per area (LMA) was excluded from the analyses because it is the reciprocal of SLA and therefore provides redundant information.

Chlorophyll content was quantified using a CCM-200 Plus chlorophyll meter (Opti-Sciences^®^, Hudson, NH, USA). Measurements were expressed as Chlorophyll Content Index (CCI) units, a relative and non-destructive estimate of foliar chlorophyll content. For each plant, three fully expanded leaves were selected, and the mean CCI value was used as the representative estimate for each individual. Readings were taken from the lower-right quadrant of each leaf, avoiding the midrib and secondary veins.

We measured three reproductive traits, namely, days to flowering, recorded as the number of days from transplanting to the opening of the first flower; height to first flower, measured from the base of the stem to the position of the first reproductive node; and total fruit number per plant recorded throughout the experiment.

### 4.4. Statistical Analyses

Continuous traits were analyzed using hierarchical analyses of variance. Domestication status (wild vs. domesticated), light treatment, and their interaction were included as fixed effects. Population or variety was nested within domestication status to account for differentiation among the three wild populations and the three domesticated varieties evaluated. The interaction between light treatment and population or variety nested within domestication status was included to evaluate heterogeneity in plastic responses among populations and varieties. Because the six groups were deliberately selected rather than randomly sampled from a broader population, inference was restricted to the populations and varieties included in this study.

Fresh leaf mass and height to first flower were log-transformed before analysis to improve residual behavior. Stem height, stem diameter, RGR, leaf area, dry leaf mass, SLA, chlorophyll content, and days to flowering were analyzed on their original scales. Leaf area and chlorophyll content were retained on their original scales to preserve biological interpretability. Leaf number was analyzed using a negative binomial model with a log link because the response was overdispersed relative to a Poisson distribution. The model included domestication status, light treatment, population or variety nested within domestication status, and their corresponding interactions. The contribution of model terms was evaluated using likelihood-ratio tests.

Fruit number was analyzed separately using a zero-inflated negative binomial model to account for overdispersion and the excess of plants that did not produce fruits, particularly under low light. The initial model included domestication status, light treatment, and their interaction as predictors of both the count and zero-inflation components. A log link was used for the count component and a logit link for the zero-inflation component. The contribution of model terms was evaluated using likelihood-ratio tests, and model fit was compared with simpler count models using the Akaike information criterion (AIC). Because the domestication status × light interaction did not significantly improve model fit, the final model retained domestication status and light treatment as additive predictors. A complementary negative binomial model evaluated the population or variety × light interaction.

The magnitude of phenotypic plasticity across environments was quantified as the rate of change of a given trait as a function of variation in light treatments using an environment-explicit approach [49,50]:

*S_αβ_* = (*z_α_* − *z_β_*)/(*x_α_* − *x_β_*)

where *z_α_* and *z_β_* are trait values and *x_α_* and *x_β_* are the corresponding light intensities. Positive and negative values indicate the direction of trait change across the light gradient. Larger absolute values indicate stronger plastic responses, whereas values close to zero indicate weak or negligible plasticity. All analyses were conducted in JMP Pro 18 (SAS Institute Inc., Cary, NC, USA). Statistical significance was evaluated at α = 0.05.

Because morphological variables are integrated into a single phenotype, differences in multivariate phenotype position across light levels were assessed using distance-based ANOVA via residual randomization permutation procedures (Procrustes ANOVA) implemented with the procD.lm function in the geomorph package for R 4.5.1. Significance was determined using 1000 permutations. Two full-factorial models were evaluated: one including domestication status, light treatment, and their interaction, and a second including accession, light treatment, and their interaction. Phenotypic Trajectory Analysis (PTA) was then used to evaluate how multivariate plastic responses unfolded across the light gradient. Trajectories were constructed by linking multivariate centroids across light levels for each group. Pairwise comparisons among trajectories were evaluated using 1000 permutations for three geometric attributes: trajectory magnitude, which quantifies total multivariate plasticity or path length; trajectory direction, which evaluates angular differences among trajectory vectors; and trajectory shape, which compares trajectory curvature and detects nonlinear or nonmonotonic responses across the environmental gradient. PTA was performed using the trajectory analysis function in geomorph.

## Figures and Tables

**Table 1:** Nested ANOVA. Population or variety was included as a fixed factor nested within domestication status. Nesting is indicated by the factor shown in parentheses, e.g., Accession (Domestication). Stem height, stem diameter, RGR, leaf area, chlorophyll content, height to first flower, and days to flowering were analyzed using 85 plants. Fresh leaf mass, dry leaf mass, and SLA were analyzed using a subsample of 54 plants with complete leaf-trait data. Number of leaves was analyzed using a nested negative-binomial model with a log link because the response variable was over-dispersed; likelihood-ratio χ^2^ statistics are reported for this trait. The number-of-leaves analysis included 86 plants with complete observations. Significant *p* values (*p* < 0.05) are shown in bold.

Trait	Source of Variation	d.f.	F or χ^2^	*p*
Stem height	Domestication status	1, 4	F = 0.09	0.7
	Light treatment	2, 8	F = 2.02	0.2
	Domestication × Light	2, 8	F = 1.21	0.3
	Accession _(Domestication)_	4, 67	F = 3.53	0.011
	Light × accession _(Domestication)_	8, 67	F = 1.97	0.06
Stem diameter	Domestication status	1, 4	F = 0.04	0.8
	Light treatment	2, 8	F = 6.15	0.024
	Domestication × Light	2, 8	F = 1.09	0.4
	Accession _(Domestication)_	4, 67	F = 2.96	0.026
	Light × accession _(Domestication)_	8, 67	F = 1.80	0.09
RGR	Domestication status	1, 4	F = 0.07	0.8
	Light treatment	2, 8	F = 1.54	0.3
	Domestication × Light	2, 8	F = 0.90	0.4
	Accession _(Domestication)_	4, 67	F = 1.80	0.1
	Light × accession _(Domestication)_	8, 67	F = 1.40	0.2
Leaf area	Domestication status	1, 4	F = 4.55	0.1
	Light treatment	2, 8	F = 17.16	0.001
	Domestication × Light	2, 8	F = 0.68	0.5
	Accession _(Domestication)_	4, 67	F = 0.66	0.6
	Light × accession _(Domestication)_	8, 67	F = 2.45	0.022
Number of leaves	Domestication status	1	χ^2^ = 0.03	0.9
	Light treatment	2	χ^2^ = 49.60	<0.001
	Domestication × Light	2	χ^2^ = 8.92	0.0116
	Accession _(Domestication)_	4	χ^2^ = 24.60	<0.001
	Light × accession _(Domestication)_	8	χ^2^ = 21.98	0.0050
SLA	Domestication status	1, 4	F = 0.12	0.7
	Light treatment	2, 8	F = 88.85	<0.0001
	Domestication × Light	2, 8	F = 0.96	0.4
	Accession _(Domestication)_	4, 36	F = 0.28	0.9
	Light × accession _(Domestication)_	8, 36	F = 1.56	0.2
Chlorophyll content	Domestication status	1, 4	F = 0.20	0.3
	Light treatment	2, 8	F = 1.54	0.272
	Domestication × Light	2, 8	F = 0.06	0.9
	Accession _(Domestication)_	4, 67	F = 10.07	<0.0001
	Light × accession _(Domestication)_	8, 67	F = 2.49	0.020
Height to first flower	Domestication status	1, 4	F = 1.86	0.2
	Light treatment	2, 8	F = 1.90	0.2
	Domestication × Light	2, 8	F = 0.78	0.5
	Accession _(Domestication)_	4, 67	F = 7.23	<0.0001
	Light × accession _(Domestication)_	8, 67	F = 1.76	0.1
Days to flowering	Domestication status	1, 4	F = 4.73	0.09
	Light treatment	2, 8	F = 3.58	0.07
	Domestication × Light	2, 8	F = 2.62	0.1
	Accession _(Domestication)_	4, 67	F = 3.28	0.016
	Light × accession _(Domestication)_	8, 67	F = 1.94	0.068

**Table 2:** Generalized count models for number of fruits. Significance was evaluated using likelihood-ratio tests.

Trait	Source of Variation	d.f.	F or χ^2^	*p*
Fruit number	Domestication status	2	18.70	<0.0001
	Light treatment	4	31.15	<0.0001
	Domestication × Light	4	2.35	0.6
	Accession (Domestication)	5	41.78	0.0001
	Light × accession (Domestication)	10	25.38	0.005

Table note. The additive zero-inflated negative binomial model provided the best fit among the models compared (AIC = 533.99). The complementary negative binomial model tested heterogeneity in fruit-number responses among accessions.

## Data Availability

Data are available in the plants platform.

## Supplementary Materials

The following supporting information can be downloaded at: https://www.mdpi.com/article/10.3390/plants15182825/s1, Table S1: Mean trait values under full, medium, and low light conditions and reaction norm slopes calculated between environments in *Capsicum annuum*.

## References

[1] Hammer K. (1984). Das Domestikationssyndrom. Die Kult..

[2] Liu W., Chen L., Zhang S., Hu F., Wang Z., Lyu J., Wang B., Xiang H., Zhao R., Tian Z. (2019). Decrease of gene expression diversity during domestication of animals and plants. BMC Evol. Biol..

[3] Andersson L., Purugganan M. (2022). Molecular genetic variation of animals and plants under domestication. Proc. Natl. Acad. Sci. USA.

[4] Darwin C. (1868). The Variation of Animals and Plants Under Domestication.

[5] Zohary D. (2004). Unconscious selection and the evolution of domesticated plants. Econ. Bot..

[6] Gepts P., Janick J. (2004). Crop domestication as a long-term selection experiment. Plant Breeding Reviews.

[7] Meyer R.S., DuVal A.E., Jensen H.R. (2012). Patterns and processes in crop domestication: An historical review and quantitative analysis of 203 global food crops. New Phytol..

[8] Milla R., Matesanz S. (2017). Growing larger with domestication: A matter of physiology, morphology or allocation?. Plant Biol..

[9] Milla R., Osborne C.P., Turcotte M.M., Violle C. (2015). Plant domestication through an ecological lens. Trends Ecol. Evol..

[10] Bradshaw A.D. (1965). Evolutionary significance of phenotypic plasticity in plants. Adv. Genet..

[11] Pigliucci M. (2001). Phenotypic Plasticity: Beyond Nature and Nurture.

[12] Schlichting C.D., Pigliucci M. (1998). Phenotypic Evolution: A Reaction Norm Perspective.

[13] Valladares F., Gianoli E., Gómez J.M. (2007). Ecological limits to plant phenotypic plasticity. New Phytol..

[14] Matesanz S., Milla R. (2018). Differential plasticity to water and nutrients between crops and their wild progenitors. Environ. Exp. Bot..

[15] Ménard L., McKey D., Mühlen G.S., Clair B., Rowe N.P. (2013). The evolutionary fate of phenotypic plasticity and functional traits under domestication in manioc: Changes in stem biomechanics and the appearance of stem brittleness. PLoS ONE.

[16] Munguía-Rosas M.A. (2022). Domestication reduces phenotypic plasticity in chaya (*Cnidoscolus aconitifolius* (Mill.) I.M. Johnst). Bot. Sci..

[17] Valladares F., Niinemets Ü. (2008). Shade tolerance, a key plant feature of complex nature and consequences. Annu. Rev. Ecol. Evol. Syst..

[18] Martínez-García J.F., Rodríguez-Concepción M. (2023). Molecular mechanisms of shade tolerance in plants. New Phytol..

[19] Pearcy R.W., Ehleringer J., Mooney H.A., Rundel P.W. (1990). Plant Physiological Ecology: Field Methods and Instrumentation.

[20] Slattery R.A., Walker B.J., Weber A.P.M., Ort D.R. (2018). The impacts of fluctuating light on crop performance. Plant Physiol..

[21] Luna-Ruiz J.J., Nabhan G.P., Aguilar-Meléndez A. (2018). Shifts in plant chemical defenses of chile pepper due to domestication. Front. Ecol. Evol..

[22] Solís-Montero V., Bello-Bedoy R., Munguía-Rosas M.A. (2023). Non-random distribution of maax pepper plants (*Capsicum annuum* var. *glabriusculum* L.) in Mayan homegardens: Impact on plant size, fruit yield and viral diseases. Agrofor. Syst..

[23] Hayano-Kanashiro C., Gámez-Meza N., Medina-Juárez L.Á. (2016). Wild pepper *Capsicum annuum* L. var. glabriusculum: Taxonomy, plant morphology, distribution, genetic diversity, genome sequencing, and phytochemical compounds. Crop Sci..

[24] Pickersgill B., Lira R., Casas A., Blancas J. (2016). Chile peppers (*Capsicum* spp.). Ethnobotany of Mexico: Interactions of People and Plants in Mesoamerica.

[25] Kraft K.H., Brown C.H., Nabhan G.P., Luedeling E., Ruiz J.J.L., d’Eeckenbrugge G.C., Hijmans R.J., Gepts P. (2014). Multiple lines of evidence for the origin of domesticated chili pepper. Proc. Natl. Acad. Sci. USA.

[26] Kraft K.H., de Jesús Luna-Ruíz J., Gepts P. (2013). A new collection of wild populations of *Capsicum* in Mexico and the southern United States. Genet. Resour. Crop Evol..

[27] Carlo T.A., Tewksbury J.J. (2014). Directness and tempo of avian seed dispersal increases emergence of wild chiltepins in desert grasslands. J. Ecol..

[28] Jiménez-Leyva A., Gutiérrez A., Ojeda-Contreras Á.J., Vargas G., Esqueda M., Orozco-Avitia J.A. (2022). Seasonal phenology, shade reliance, and ecophysiology of wild *Capsicum annuum* var. *glabriusculum* in the Sonoran Desert. J. Arid Environ..

[29] Hernández-Verdugo S., Luna-Reyes R., Oyama K. (2015). Genetic structure and differentiation of wild and domesticated populations of *Capsicum annuum* (Solanaceae) in Mexico. Plant Syst. Evol..

[30] Jiménez-Leyva A., Orozco-Avitia J., Gutiérrez A., Vargas G., Sánchez E., Muñoz E., Esqueda M. (2022). Functional plasticity of *Capsicum annuum* var. *glabriusculum* through multiple traits. AoB Plants.

[31] Rai S., Basfore S., Rai S., Rai U., Mukhia D.J., Sikder S., Medda P.S. (2025). Effect of different growing environments on growth, yield and quality of bell pepper (*Capsicum annuum* L. var. *grossum* Sendt.) genotypes under terai region of West Bengal. J. Appl. Hortic..

[32] McCoy J.E., McHale L.K., Kantar M., Jardón-Barbolla L., Mercer K.L. (2022). Environment of origin and domestication affect morphological, physiological, and agronomic response to water deficit in chile pepper (*Capsicum* sp.). PLoS ONE.

[33] De la Cruz-Ricardez D., Lagunes-Espinoza L.C., Ortiz-García C.F., Hernández-Nataren E., Soto-Hernández R.M., Acosta-Pech R.G. (2023). Phenology, yield, and phytochemicals of *Capsicum* spp. in response to shading. Bot. Sci..

[34] Serrano-Mejía C., Bello-Bedoy R., Arteaga M.C., Castillo G.R. (2022). Does domestication affect structural and functional leaf epidermal traits?. Plants.

[35] Doebley J.F., Gaut B.S., Smith B.D. (2006). The molecular genetics of crop domestication. Cell.

[36] Sultan S.E., Pfennig D.W. (2021). Phenotypic plasticity as an intrinsic property of organisms. Phenotypic Plasticity & Evolution: Causes, Consequences, Controversies.

[37] Des Marais D.L., Hernandez K.M., Juenger T.E. (2013). Genotype-by-environment interaction and plasticity: Exploring genomic responses of plants to the abiotic environment. Annu. Rev. Ecol. Evol. Syst..

[38] Liu Y., Dawson W., Prati D., Haeuser E., Feng Y., van Kleunen M. (2016). Does greater specific leaf area plasticity help plants to maintain a high performance when shaded?. Ann. Bot..

[39] Solís-Montero V., Martínez-Natarén D.A., Parra-Tabla V., Ibarra-Cerdeña C., Munguía-Rosas M.A. (2020). Herbivory and anti-herbivore defences in wild and cultivated *Cnidoscolus aconitifolius*: Disentangling domestication and environmental effects. AoB Plants.

[40] De Kroon H., Huber H., Stuefer J.F., Van Groenendael J.M. (2005). A modular concept of phenotypic plasticity in plants. New Phytol..

[41] Stotz G.C., Salgado-Luarte C., Escobedo V.M., Gianoli E. (2025). Phenotypic integration limits the variation in plant phenotypic plasticity among traits: A meta-analysis. Funct. Ecol..

[42] Forsman A. (2015). Rethinking phenotypic plasticity and its consequences for individuals, populations and species. Heredity.

[43] van Kleunen M., Fischer M. (2005). Constraints on the evolution of adaptive phenotypic plasticity in plants. New Phytol..

[44] Lorenzo C.D., Alonso Iserte J., Sanchez Lamas M., Antonietti M.S., Garcia Gagliardi P., Hernando C.E., Dezar C.A.A., Vazquez M., Casal J.J., Yanovsky M.J. (2019). Shade delays flowering in *Medicago sativa*. Plant J..

[45] Donohue K., Messiqua D., Hammond-Pyle E., Heschel M.S., Schmitt J. (2000). Evidence of adaptive divergence in plasticity: Density- and site-dependent selection on shade-avoidance responses in *Impatiens capensis*. Evolution.

[46] Steinger T., Roy B.A., Stanton M.L. (2003). Evolution in stressful environments II: Adaptive value and costs of plasticity in response to low light in *Sinapis arvensis*. J. Evol. Biol..

[47] Wang M.-Z., Li H.-L., Liu C.-X., Dong B.-C., Yu F.-H. (2022). Adaptive plasticity in response to light and nutrient availability in the clonal plant *Duchesnea indica*. J. Plant Ecol..

[48] Alon E., Shapira O., Azoulay-Shemer T., Rubinovich L. (2022). Shading nets reduce canopy temperature and improve photosynthetic performance in ‘Pinkerton’ avocado trees during extreme heat events. Agronomy.

[49] Morrissey M.B., Liefting M. (2016). Variation in reaction norms: Statistical considerations and biological interpretation. Evolution.

[50] Gómez J.M., Perfectti F., González-Megías A., Armas C. (2025). Quantifying phenotypic plasticity: A call for consistency. Funct. Ecol..

