# Supplementary material for: Phenotypic Plasticity Beyond Domestication: Trait- and Accession-Dependent Responses to Light in Capsicum annuum
Source: Plants (Basel). 2026 Sep 15;15(18):2825. doi: 10.3390/plants15182825 (PMC13610071; doi:10.3390/plants15182825)
Supplement: Supplementary file 1 [file plants-15-02825-s001.zip › plants-4483426-supplementary.pdf]

Table S1. Mean trait values under full, medium, and low light conditions and reaction norm slopes calculated between environments in *Capsicum annuum*.

Trait	Full	Moderated	Low	S (F–M)	S (M–L)	S (F–L)
Stem height	9.80	12.56	10.75	-0.0063	0.0045	-0.0011
Stem diameter	4.21	4.81	3.40	-0.0014	0.0035	0.0010
Number of leaves	78.72	61.40	33.15	0.0398	0.0698	0.0543
RGR	0.139	0.157	0.151	-0.0000	0.0000	-0.0000
Leaf area	8.95	11.31	22.24	-0.0054	-0.0270	-0.0158
SLA	201.01	211.19	426.36	-0.0234	-0.5313	0.2683
Chlorophyll	25.33	26.89	19.01	-0.0036	0.0194	0.0075
Height to first flower	34.76	39.53	46.53	-0.0109	-0.0173	-0.0140
Days to flower	47.54	48.53	60.07	-0.0023	-0.0285	-0.0149
Fruit number	25.11	22.33	1.93	0.0064	0.0504	0.0276

Full = 1000 μmol m^{-2} s^{-1}, Medium = 565 μmol m^{-2} s^{-1}, and Low = 160 μmol m^{-2} s^{-1}. Reaction norm slopes were calculated as $S_{a\beta} = (z_a - z_\beta)/(x_a - x_\beta)$, where z_a and z_β are mean trait values and x_a and x_β are the corresponding light intensities. Positive and negative values indicate the direction of trait change across the light gradient, and larger absolute values indicate stronger plastic responses.
